# Machine Learning Techniques for THz Imaging and Time-Domain Spectroscopy

**DOI:** 10.3390/s21041186

**Published:** 2021-02-08

**Authors:** Hochong Park, Joo-Hiuk Son

**Affiliations:** 1 Department of Electronics Engineering, Kwangwoon University, Seoul 01897, Korea; hcpark@kw.ac.kr; 2 Department of Physics, University of Seoul, Seoul 02504, Korea

**Keywords:** terahertz imaging, terahertz time-domain spectroscopy, machine learning, classification, regression, supervised learning, feature extraction

## Abstract

Terahertz imaging and time-domain spectroscopy have been widely used to characterize the properties of test samples in various biomedical and engineering fields. Many of these tasks require the analysis of acquired terahertz signals to extract embedded information, which can be achieved using machine learning. Recently, machine learning techniques have developed rapidly, and many new learning models and learning algorithms have been investigated. Therefore, combined with state-of-the-art machine learning techniques, terahertz applications can be performed with high performance that cannot be achieved using modeling techniques that precede the machine learning era. In this review, we introduce the concept of machine learning and basic machine learning techniques and examine the methods for performance evaluation. We then summarize representative examples of terahertz imaging and time-domain spectroscopy that are conducted using machine learning.

## 1. Introduction

Terahertz (THz) imaging and THz time-domain spectroscopy (THz-TDS) are powerful tools for the characterization of test samples based on their unique resonance properties in the THz frequency range. In recent years, numerous THz applications have been developed in biomedical and engineering fields [1,2,3,4,5,6,7]. In particular, they can be used for disease diagnosis in biological samples [8,9,10,11,12,13,14,15,16,17,18,19,20], characteristic analysis of biological samples [21,22,23,24,25,26] and biomolecules [27,28,29,30,31,32,33], non-destructive detection of artifacts and objects in samples [34,35,36,37,38,39,40,41], identification of hidden materials and their properties [42,43,44,45], estimation of the components in a mixture [46,47,48,49,50,51], and so on. Due to the rapid evolution of THz technologies and their increased availability, it is expected that the application of THz imaging and THz-TDS will expand.

The acquired THz signals from test samples, which include one-dimensional (1-D) time-domain signals and two-dimensional (2-D) spatial-domain images by transmission or reflection, contain information about the samples at specific frequencies. By appropriate analysis of the acquired signals, we can extract important embedded information that serves as a signature of a feature of interest. In a broad sense, any task that analyzes a signal for extracting embedded information, whatever its specific objective, can be regarded as a potential application of artificial intelligence, such as speech recognition [52,53,54,55,56,57] and image recognition [58,59,60,61,62,63].

The conventional methods for early artificial intelligence generally adopted an analytical approach that describes a mathematical representation of an optimization problem and searches for the solution under certain constraints, which is called a knowledge-based approach [64]. For most tasks, however, the problems are not well described using mathematical rules because the main tasks are often abstract and not suited to specific step-by-step processes. For example, although humans can easily distinguish between dogs and cats without being aware of the process involved, it is not easy to define mathematical rules that describe the differences between dogs and cats.

To address the limitations of knowledge-based approach, a new strategy that mimics human processes has been developed. Humans can perform a certain task after repeatedly executing the same task while being guided by feedback depending on their decisions or actions. For example, humans can distinguish between dogs and cats because they have seen many examples of these animals and possess pertinent information that allows for the discrimination of the two species based on daily activities and observation. As such, they can perform this task by learning through experiences, not by studying the rules that describe this task. This approach is called a learning-based approach, as opposed to a knowledge-based approach [64]. Analogously, we can develop machines to perform a given task using a learning-based approach, and this process is called machine learning (ML). In ML, the learning process is expressed by mathematical equations, and the “experience” for the task is provided in the form of a training dataset, wherein each constituent element is called an instance. The key to ML is learning a task using training data, and not the explicit determination of task functions.

As the performance of ML has improved, this technique has been widely used in THz applications to analyze the information in acquired THz signals for given tasks. In this review, we introduce basic ML techniques in their most general form and describe the issues to consider when applying ML to the tasks with certain constraints. In addition, we explain the statistical methods used to evaluate the performance of tasks. Finally, we examine the nature of THz applications from an ML perspective, compared with the applications with natural signals, and summarize representative examples of ML-based THz imaging and THz-TDS. These examples were selected to cover a variety of ML techniques that are applied to the various tasks such as disease diagnosis, disease level estimation, component analysis, and material identification.

## 2. Machine Learning Techniques

### 2.1. Overview

#### 2.1.1. Categories of Machine Learning

In general, ML is divided into three categories. One category is supervised learning, which uses a training dataset consisting of many inputs and their labels [65,66]. Each label represents a target output for each input. In supervised learning, a model is learned that best describes the input-label relationship in the training dataset. For example, the task of dog/cat discrimination is an example of supervised learning, and each instance consists of an image of a dog or cat and its label. This review deals only with topics in supervised learning because most tasks of recognition and estimation fall in this category.

The other two categories of ML are unsupervised learning and reinforcement learning [65]. Unsupervised learning uses a training dataset without labels and learns the data properties embedded in the inputs [67,68,69,70,71]. Reinforcement learning has no training dataset and learns the desired model using a trial-and-error approach; it is usually used when learning how to play a game [72,73,74,75].

#### 2.1.2. Classification and Regression

Two common tasks of supervised learning are classification and regression [65]. Classification maps the input onto one predefined class. In this case, an instance in the training dataset consists of an input and its class as a label, where each label is usually expressed as an integer. Most tasks of detection and recognition, such as dog/cat discrimination, correspond to classification.

Regression maps the input to a numeric value of the floating-point type, which is generally considered an estimation task. In regression, an instance in the training dataset consists of input-value pairs. For example, we can estimate the extent of disease progression from medical images using values ranging from 0.0 (normal) to 1.0 (severe). Regression tasks can be converted to classification tasks by defining discrete ranges of values and assigning each to a class, by sacrificing the resolution of the estimated values.

#### 2.1.3. Overfitting and Generalization

The training dataset for the task is a set of sampled instances from an infinite number of all possible instances for a task. We then assume that the training dataset represents the overall properties of all possible instances and that a generalized model for the task can be learned using only the training dataset. In reality, however, this assumption is not true, and the learned model tends to fit specific properties observed only in the training dataset, which is called overfitting in learning [76]. Another aspect of overfitting is related to noise in the training data; ML might learn noise properties as well as signal properties. In THz applications, for example, because the task usually uses private data acquired in each individual laboratory, it is difficult to obtain sufficient training data required for representing general model and overfitting is prone to occur. To reduce overfitting and enhance generalization, various techniques have been developed, such as regularization [77,78], drop-out [79,80], and ensemble learning [81].

**Figure 1:**
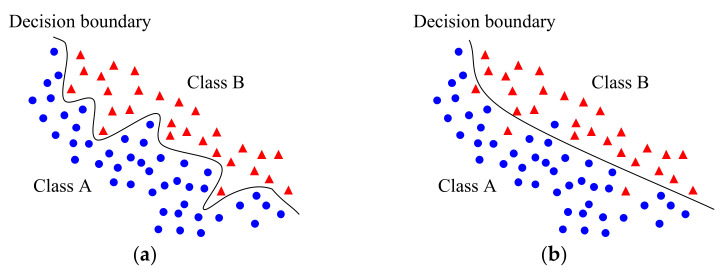
Example of overfitting in classification. (**a**) Decision boundary that best fits training data; (**b**) Generalized decision boundary that facilitates improved performance in real-world applications.

Figure 1 shows an example of overfitting in binary classification, where the symbols represent 2-D inputs in the training dataset. Figure 1a shows the learned decision boundary with the best fit, but it seems to be incorrect, considering the presence of noise in the training data. Instead, a simpler boundary as shown in Figure 1b could be a generalized boundary that facilitates improved performance in real-world applications, although some inputs in the training dataset are misclassified.

Considering overfitting in ML, we should evaluate the performance of the learned model using instances that are not included in the training dataset; a set of these instances for performance evaluation is called a testing dataset. If we observe high performance for the training dataset but low performance for the testing dataset, the learned model has poor generalization and does not yield high performance in real-world applications. In general, we first generate a set of instances that describe a task, then arbitrarily divide the set into training and testing datasets.

Although the size of the training dataset plays a key role in controlling overfitting, there are no practical rules for choosing it. In theory, the Vapnik–Chervonenkis (VC) dimension measures the capacity of learning model, which serves as a guideline for choosing the size of the training dataset [82]. In practice, however, the VC dimension is difficult to calculate, and the size of the training dataset is usually determined empirically by analyzing the learning behavior and performance of ML model in use [83].

### 2.2. Feature Extraction and Reduction

Although the originally acquired signals contain raw input information, we do not typically use them as inputs in their original form. The raw signals contain significant redundancy with respect to the task, and the use of ML to analyze raw signals becomes inefficient due to the interfering effect of redundant components. Hence, we first extract key features that better represent core information from the raw signals and use them as inputs of both the training and testing datasets. As such, ML learns a model that best describes a function from input features to targets in the training dataset. 

The number of extracted features is usually less than the number of samples in the raw signal. In addition to improved ML performance, we can then reduce the computational load of learning and the complexity of the learned model. The choice of features depends heavily on the task, and the extraction of optimal features is a key factor of high performance. ML based on raw signals instead of features is also widely used, especially in image analysis [59,62].

After extracting the features, we can measure the relevance of each feature to a given task and delete less relevant features in order to further reduce the number of features as much as possible. This procedure is called feature reduction. When the number of training data is small, feature reduction helps enhance generalization in learning by enabling to use a small learning model. Three common methods for feature reduction are described.

#### 2.2.1. Principal Component Analysis

**Figure 2:**
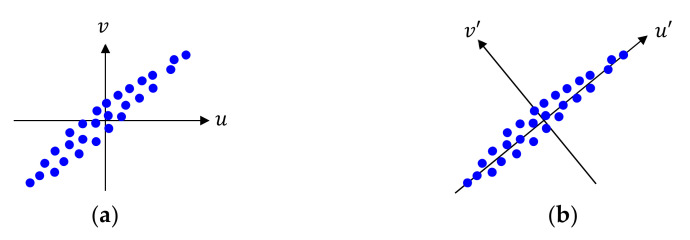
Operation of principal component analysis (PCA) in a 2-D space. (**a**) Data distribution in the original domain; (**b**) Data distribution in the transformed domain by PCA.

The principal component analysis (PCA) is a mathematical tool for transforming high-dimensional data to lower-dimensional data with minimal information loss, by using the dependencies between the variables [67,70]. We consider 2-D input features $\left(u_{n},v_{n}\right)$ with a zero mean, as shown in Figure 2a. They have similar variance along each axis, and both $u_{n}$ and $v_{n}$ play a role in determining the feature properties. If we apply a transform to $\left(u_{n},v_{n}\right)$ onto another 2-D domain such that new 2-D features $\left(u_{n}^{\prime },v_{n}^{\prime }\right)$ in the transformed domain have different variances along two axes, as shown in Figure 2b; this transform is simply a rotation. Then, 1-D features $\left(u_{n}^{\prime }\right)$ can represent key properties of 2-D features $\left(u_{n}^{\prime },v_{n}^{\prime }\right)$ with minimal loss, and $u_{n}^{\prime }$ is more important than $v_{n}^{\prime }$ in representing the feature properties. The u′-axis with the largest variance is called the first principal component, and the v′-axis is called the second principal component. Thus, PCA is a process to compute the principal components of data.

When the training dataset consists of N instances and the number of extracted features is $M_{0}$, the input features in each instance can be denoted by a $M_{0}$-dimensional vector $X_{n}$, 1≤n≤N. The principal components of the features can then be computed using eigenvalue decomposition of $X_{n}$. From a $M_{0}\times M_{0}$ covariance matrix of $X_{n},$ its eigenvectors correspond to the principal components and its eigenvalues correspond to the variances along the associated principal components. With $M<M_{0}$, we compute M principal components with the M largest eigenvalues and obtain M features by computing the projection of $X_{n}$ to each of the M principal components.

For better feature reduction, we can apply a high-dimensional function Q to $X_{n}$ and apply PCA to $Q(X_{n}).$ In practice, because of the high computational load caused by the high dimension of Q, we use a kernel approach called kernel-PCA, rather than directly computing the principal components of $Q(X_{n})\;$[84,85]. The major kernel functions κ for two vectors are defined as follows, where d, α, and δ are the function parameters.

- polynomial kernel: $\kappa \left(a,b\right)={\left(1+a^{T}b\right)}^{d}$
- Gaussian kernel: $\kappa \left(a,b\right)=\exp(-\alpha {\left|a-b\right|}^{2})$
- hyperbolic tangent kernel: $\kappa \left(a,b\right)=\tan h\left(a^{T}b+\delta \right)$

After computing $\kappa \left(X_{i},X_{j}\right)$ for pairs of feature vectors and solving a given matrix equation, we can compute the principal components of $Q(X_{n})$, without explicitly computing $Q(X_{n})$; its detail can be found in [85].

PCA can also be used to identify outliers in the training dataset for classification, which helps improve ML performance by removing erroneous information in noisy data. Specifically, for each class, the training data that are far from their centroid in the first few principal components are declared outliers [44].

#### 2.2.2. Relief and ReliefF

The Relief algorithm is a method for determining important features in binary classification [86]. It is based on the idea that, for better classification, the feature vectors for each class should form a distinct cluster and the two classes should be sufficiently separated in the feature space. This requirement is satisfied if the features have similar values for the same class and different values for different classes.

In Relief, for a given feature vector $X_{n}$, we determine its two nearest neighbors $H_{n}$ and $M_{n}$, one from the same class (hit) and the other from different class (miss). Then, for the m-th feature element in $X_{n}$ denoted as $x_{mn}$, $1\leq m\leq M_{0}$, we update its weight $w_{m}$ as shown in Equation (1) for all m. By repeating this update by randomly selecting $X_{n},$ $w_{m}$ converges to the degree of feature relevance to classification, and we select M features with the M largest $w_{m}$.

(1) $$w_{m}\leftarrow w_{m}+\frac{\left|x_{mn}-m^{th}\;element\;in\;M_{n}\right|-\left|x_{mn}-m^{th}element\;in\;H_{n}\right|}{\max\left(x_{mn}\right)-\min\left(x_{mn}\right)}$$

For multi-class classification, Relief is extended to ReliefF [86]. Similar to Relief, we randomly select $X_{n}$ and search for its R nearest neighbors from the same class and R nearest neighbors from each of the different classes. Based on an idea similar to Equation (1), we update the feature weight by computing the differences in the feature values for the same class and different classes.

#### 2.2.3. Maximal Information Coefficient

The maximal information coefficient (MIC) is a method for measuring data relationship based on mutual information between data [87]. In particular, MIC can determine the degree of data relationship when data are correlated in various and complex ways, which is not possible using the conventional Pearson correlation coefficient [88]. Intuitively, important features correspond to those with a high correlation to the target; hence, MIC can serve as a measure of feature importance.

**Figure 3:**
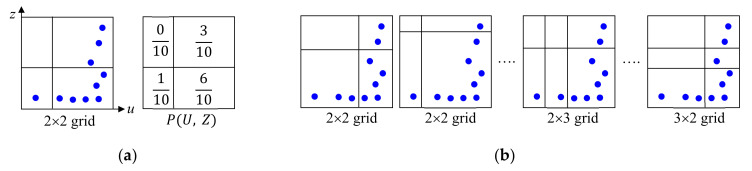
Example of computing maximal information coefficient (MIC). (**a**) 2×2 grid and probability mass function; (**b**) Grids with different sizes and shapes.

We explain how to compute the MIC of $u_{i}$ and $z_{i}$ using the example shown in Figure 3, where the symbols represent $\left(u_{i},\;z_{i}\right)$. As in Figure 3a, we apply a 2×2 grid to the domain of $\left(u_{i},\;z_{i}\right)$ and compute a 2-D probability mass function (PMF) denoted by $P\left(U,Z\right)$, where U and Z are discrete random variables, by counting the number of $\left(u_{i},\;z_{i}\right)$ in each grid box. Then, we compute the mutual information $I\left(U;Z\right)$ induced on this grid using Equation (2), where $P\left(U\right)$ and $P\left(Z\right)$ are the marginal PMF of each variable and $D_{KL}$ is the Kullback–Leibler divergence [89].

(2) $$I\left(U;Z\right)=D_{KL}(P\left(U,Z\right)|P\left(U\right)P\left(Z\right))$$

We repeat this process for different 2×2 grid shapes as in Figure 3b and determine the maximum $I\left(U;Z\right)$ for all possible 2×2 grid shapes, which is denoted by $MIC_{0}\left(2,\;2\right)$. Next, we compute $MIC_{0}\left(a,\;b\right)$ for different values of a and b as in Figure 3b and find the global maximum of $MIC_{0}\left(a,\;b\right)$ across all a and b after normalization by log(min(a,b)), which gives the final MIC of $\left(u_{i},\;z_{i}\right)$. After computing the MIC of (feature, target) for each feature independently, we select M features with the M largest MIC. In ML-based THz-TDS, MIC is widely used to determine the important frequencies in input spectral information for a given task [16,18].

### 2.3. Models of Machine Learning

For each instance in the training dataset, the M input features after feature reduction are denoted by the M-dimensional vector $X_{n}$ and L targets are denoted by the L-dimensional vector $Y_{n}$, 1≤n≤N, where N is the number of instances. The m-th feature element in $X_{n}$ is denoted by $x_{mn}$, 1≤m≤M, and the *l*-th target element in $Y_{n}$ is denoted by $y_{ln}$, 1≤l≤L. Then, the entire training dataset becomes a $\left(M+L\right)\times N$ matrix.

In classification, there are two possible ways to choose the number of target elements L. One way is to have one target element (L=1) with different values for different classes. For example, in binary classification with two classes, each usually being called positive and negative, the target value is one for positive and zero for negative. The other way is to have individual target element for each class; for example, in three-class classification, L=3 and $Y_{n}$ of each class becomes either ${\left[1\;0\;0\right]}^{T}$, ${\left[0\;1\;0\right]}^{T}$, or ${\left[0\;0\;1\right]}^{T}$. In regression, L is the number of values to be estimated.

**Figure 4:**
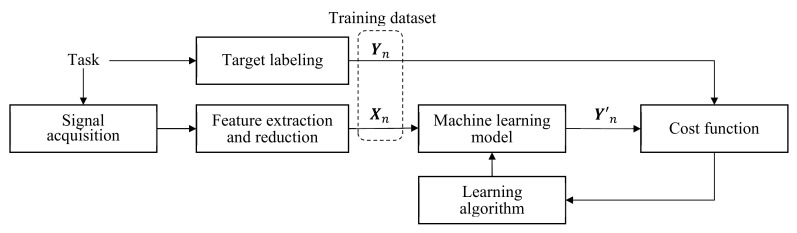
Process of model learning in typical machine learning.

The model output for input $X_{n}$ is denoted by $Y_{n}^{\prime }$, and a cost function $C\left(Y_{n},Y_{n}^{\prime }\right)\geq 0$ is defined that measures the error between $Y_{n}^{\prime }$ and its target $Y_{n}$. Then, the average cost $\mathbb{C}=\frac{1}{N}\overset{N}{\underset{n=1}{\sum }}C\left(Y_{n},Y_{n}^{\prime }\right)$ is used to measure the overall model quality with respect to the training dataset. Model learning is a process that determines the model parameters using a learning algorithm such that ℂ is minimized, as shown in Figure 4. We describe various learning models and learning algorithms.

#### 2.3.1. Neural Network

**Figure 5:**
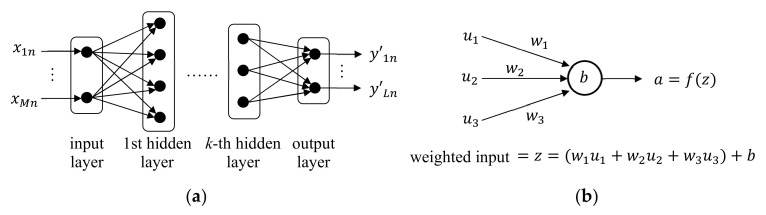
Structure and operation of neural network. (**a**) Layered structure of neurons; (**b**) Operation of each neuron in hidden and output layers.

A neural network (NN) is a learning model that mimics the human neural system [66,90,91]. NN consists of many neurons as shown in Figure 5a. Multiple neurons constitute a layer, and the layers are connected in cascade with one input layer, any number of hidden layers, and one output layer. When the number of hidden layers is two or larger, it is usually called a deep neural network (DNN). In a typical NN, all neurons in the adjacent layers are all connected as shown in Figure 5a, and this layer structure is called a fully-connected layer.

The input layer has M neurons, each accepting $x_{mn}$, and the output layer has L neurons, each yielding $y\prime_{ln}$. Each neuron in the hidden and output layers accepts multiple inputs from neurons in the previous layer. Then, as shown in Figure 5b, the neuron output a is computed by applying an activation function $f\left(\cdot \right)$ to a weighted input z, where z is computed using weights $w_{i}$ assigned to the connections and bias b assigned to the neuron. The common activation functions are defined as follows [92,93,94,95].

- sigmoid: $f\left(z\right)=\frac{1}{1+\exp\left(-z\right)}$
- hyperbolic tangent: $f\left(z\right)=\tan h\left(z\right)$
- softmax: $f\left(z_{l}\right)=\frac{\exp\left(z_{l}\right)}{\sum_{j=1}^{L}\exp\left(z_{j}\right)},\;1\leq l\leq L$
- rectified linear unit (ReLU): $f\left(z\right)=\left\{\begin{array}{c} 0,\;z<0\\ z,\;z\geq 0 \end{array}\right.$

In NN for classification with each output neuron associated with one class, the output neuron usually uses softmax activation, wherein $z_{l}$ is a weighted input in the l-th neuron. Then, the l-th output neuron determines the probability that the input is likely to be the l-th class, and the class with the highest probability is selected. In binary classification into positive and negative classes, the output neuron usually uses sigmoid activation. Then, with a given threshold λ, the input is classified as positive if the output is larger than λ; otherwise, it is classified as negative. In regression, each output neuron corresponds to each value to be estimated, and sigmoid or hyperbolic tangent activation is usually used.

The learning of NN is a procedure to determine all NN parameters such as weights and biases. Instead of computing the optimal parameters by solving the equations, we iteratively update the parameters such that ℂ decreases at each iteration; each update corresponds to one learning step. For this procedure, we measure the gradient of ℂ with respect to each parameter and update the parameter slightly in a negative gradient direction, which guarantees a reduction in ℂ. This gradient-based update is called the gradient descent (GD) method [96,97]. A structured method for computing gradients for all parameters, called backpropagation, has been developed [98,99]. It successively determines gradients from output to input layers, considering that gradients in the output layer are easily computed and gradients in adjacent layers have a closed-form relationship in the backward direction. Many variants of GD and learning techniques for faster learning of NN have been developed [100,101,102,103,104,105].

If the number of training data is not large enough, compared with the number of NN parameters, there is a risk of yielding poorly learned NN with severe overfitting. In practice, when the training dataset is given, we need to compare the performance of many NNs of different structures and select the final one with acceptable performance.

#### 2.3.2. Convolutional Neural Network

**Figure 6:**
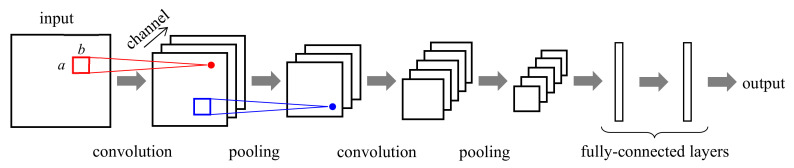
Structure and operation of convolutional neural network (CNN).

A convolutional neural network (CNN) is a specialized NN structure for 2-D images, wherein each image pixel is associated with one neuron [106,107,108]. In the CNN, as shown in Figure 6, we apply a 2-D convolution with a small receptive field of size a×b, say 3×3 or 5×5, to the input image and compute the output image that represents the local characteristics of the input image. By applying multiple convolutions with different convolution filters in parallel, we obtain multiple output images, each referred to as a channel or feature map. Each channel represents its modeling result of input for different aspects. For example, in Figure 6, we apply three convolution filters to the input and have output with three channels. We then apply pooling to each channel and compute the maximum or average of the pixel values within the local area. The purpose of pooling is to increase modeling invariance to a small change in the signals [108]. We connect a layer consisting of one convolution stage and one pooling stage in cascade to build a larger CNN. In general, we connect a few fully-connected layers to the end of the CNN layers so that we collect the modeling results of the CNN layers and determine the final output. The CNN parameters such as the filter coefficients are also determined using the backpropagation. In CNN, we usually use raw input images, instead of features.

#### 2.3.3. Decision Tree

**Figure 7:**
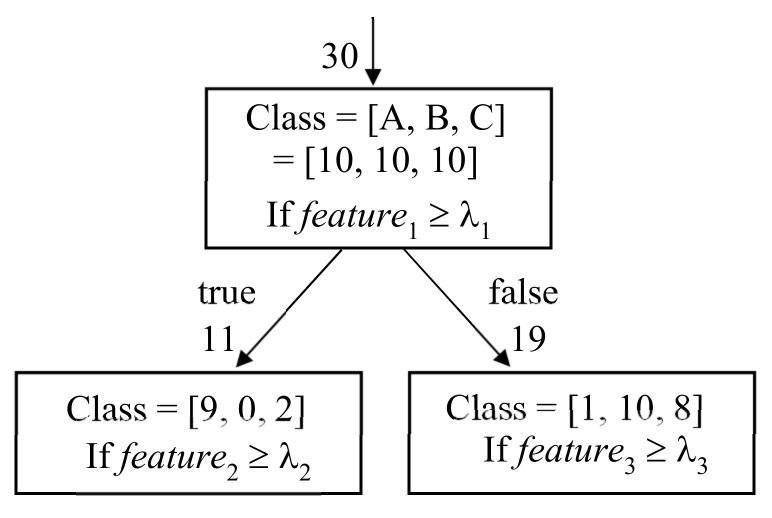
Example of operation at the node in decision tree for 3-class classification.

A decision tree is a learning model that performs iterative binary tree searches, wherein each tree node performs one binary decision [109,110]. In classification, we choose one feature element and its threshold λ at a node and split all input instances into two subsets of instances by checking if the given feature element for the input instances is larger than λ. Figure 7 shows an example of operation at the node in a decision tree for three-class classification into A, B, and C. The node has 30 input instances with 10 instances in each class. A feature element $feature_{1}$ and threshold $\lambda_{1}$ are assigned to this node. Among the 30 input instances, nine instances in class A and two instances in class C have $feature_{1}$ larger than $\lambda_{1}$, and the remaining 19 instances have $feature_{1}$ smaller than $\lambda_{1}$. In this way, two nodes are generated, each with 11 and 19 input instances, and the class population is changed at each node. We build many layers of nodes by repeating the same operation in each output node with a new feature element and threshold and obtain the required model for classification.

The key aspect of the decision tree is to select the feature element and its threshold for use at the appropriate active node. We first define an impurity metric of the node as follows, where $p_{c}$ is the ratio of instances in class *c* among all input instances to the node [111].

- Gini impurity: $G=1-\overset{C}{\underset{c=1}{\sum }}p_{c}^{2}$
- entropy impurity: $H=1-\overset{C}{\underset{c=1}{\sum }}p_{c}\log_{2}\left(p_{c}\right)$ for $p_{c}\neq 0$

For example, the left output node in Figure 7 has Gini impurity of $1-{\left(\frac{9}{11}\right)}^{2}-{\left(\frac{0}{11}\right)}^{2}-{\left(\frac{2}{11}\right)}^{2}$. Then, for all combinations of the feature element, threshold, and active node, we search for the combination that yields the two purest output nodes with the minimum cost defined in Equation (3), where $d_{true}$ and $d_{false}$ are the number of instances of the two output nodes and $im_{true}$ and $im_{false}$ are their impurities [111].

(3) $$cost=\frac{d_{true}}{d_{true}+d_{false}}im_{true}+\frac{d_{false}}{d_{true}+d_{false}}im_{fasle}$$

Because we build the decision tree by sequentially splitting the output nodes, we can control overfitting by properly deciding the tree depth and the number of nodes using pre-pruning or post-pruning. In general, therefore, the decision tree is suitable for tasks with small training dataset, compared with more complex models such as NN.

#### 2.3.4. k-Nearest Neighbor

A k-nearest neighbor (kNN) is a method for classification with no explicit training stage [112]. When classifying an input, we search for its *k* nearest neighbors in the training dataset using a given distance metric. Then, we identify each class of the *k* nearest neighbors and classify the input into the majority class among them. The most common distance metrics include the Euclidean distance and cosine distance.

#### 2.3.5. Support Vector Machine

**Figure 8:**
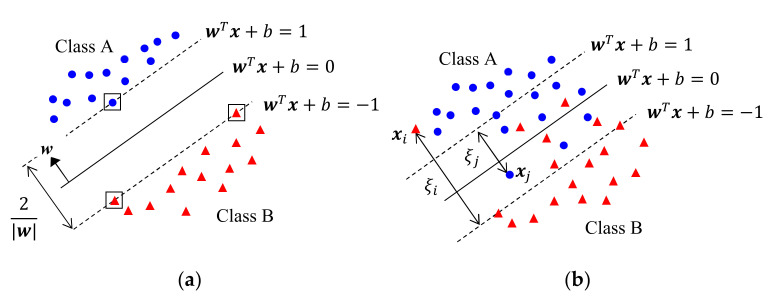
Operation of support vector machine (SVM) in a 2-D space. (**a**) Separable case; (**b**) Non-separable case.

The concept of support vector machine (SVM) involves dividing the data into two classes using a hyperplane as the decision boundary [113,114]. Its operation in a 2-D space is explained in Figure 8, wherein the symbols represent the 2-D $X_{n}$ of two classes. Figure 8a corresponds to a perfectly separable case, in which we want to find the decision boundary for two conditions: (i) a decision boundary with no misclassification and (ii) the maximal separation margin between two classes.

Let a vector in 2-D space be $x={\left[x_{1}\;x_{2}\right]}^{T}$ and the equation of the hyperplane be $w^{T}x+b=0$, where w and b are parameters to be determined. We set the two margins to be $w^{T}x+b=1$ and $w^{T}x+b=-1$. Then, w is normal to the hyperplane and the distance between the two margins becomes $2/\left|w\right|$, as shown in Figure 8a. We also set the target $y_{n}$ of each class to be +1 and −1. Then, the condition for w and b becomes $y_{n}\left(w^{T}X_{n}+b\right)\geq 1$, subject to minimum $\left|w\right|$, and we can compute w and b by solving Equation (4) using a Lagrange multiplier $\eta_{n}\geq 0$ [113]. w is determined only from data on the margin that satisfies $y_{n}\left(w^{T}X_{n}+b\right)=1$, which are called support vectors; they are identified with boxes in Figure 8a.

(4) $$\frac{1}{2}{\left|w\right|}^{2}-\overset{N}{\underset{n=1}{\sum }}\eta_{n}\left[y_{n}\left(w^{T}X_{n}+b\right)-1\right]$$

When $X_{n}$ are not separable with a hyperplane as in Figure 8b, we must use soft SVM, which allows error with a minimum distance to the margin. The details of soft SVM can be found in [113]. For a non-separable case, we can transform $X_{n}$ to another domain and apply SVM to the transformed $X_{n}$, which is called a kernel SVM [113].

The operation of SVM can be modified for regression, resulting in support vector regression (SVR) [115]. Similar to SVM, SVR depends only on a subset of the training dataset because $X_{n}$ that has a low prediction error has little influence in determining the predictor model. In SVR, we search for w and b using Equation (5), and all the prediction errors should be within the range from −ε to ε.

(5) $$\mathrm{Minimize}\;\frac{1}{2}{\left|w\right|}^{2},\;\mathrm{subject}\;\mathrm{to}\;\left|y_{n}-w^{T}X_{n}-b\right|<\varepsilon$$

#### 2.3.6. Ensemble Learning

Ensemble learning is a strategy to achieve better generalization in ML [81]. The idea is to learn multiple models independently for a single task and to merge all the information from the models to make the final decision, with the intent of improving generalization by an averaging effect. In this strategy, we should use models with different properties so that each has different types of learning artifacts. Although ensemble learning increases the learning complexity due to multiple learning processes, it is widely used for the tasks where it is difficult to obtain many training data.

**Figure 9:**
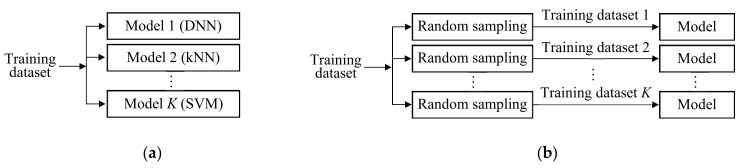
Structures of ensemble learning. (**a**) Different learning methods for each model with the same training dataset; (**b**) Different training datasets for each model with the same learning method.

We generate independent models by randomly sampling learning methods and/or training data from their candidates. The random sampling with replacement is called bootstrap aggregating (in short, bagging) [116], and that without replacement is called pasting [117]. Figure 9 shows two simple structures of ensemble learning. One structure uses models with different learning methods, such as DNN, kNN, and SVM, but the same training dataset. The other structure uses different training datasets for the same learning method.

In the testing stage, we apply the input to all models simultaneously and obtain their outputs. In classification, we choose the majority class among all outputs, which is called hard voting. Alternatively, we choose the class with the highest average probability over all the models, assuming that the class probability is available at the output; this is called soft voting. In regression, we usually select the average of all outputs as the final estimated value. Instead of using a fixed method for the final decision, we can use another leaning model for this purpose, which is called stacked generalization (in short, stacking) [118].

#### 2.3.7. Random Forest

One drawback of the decision tree is that the learned result depends heavily on the order of applying feature elements to the nodes. Random forest is an ensemble learning model consisting of multiple decision trees with the structure shown in Figure 9b, thereby yielding more generalized model by averaging the learned results of multiple decision trees [119,120].

Because random forest uses only a decision tree, different training datasets are applied to each decision tree based on a bagging approach. When building a tree, the regular decision tree searches for the best feature element among all feature elements. In random forest, however, in order to incorporate randomness into the decision tree for more generalization, it searches for the best feature element among those that are randomly sampled from all feature elements using a bagging approach.

#### 2.3.8. Adaptive Boosting

**Figure 10:**
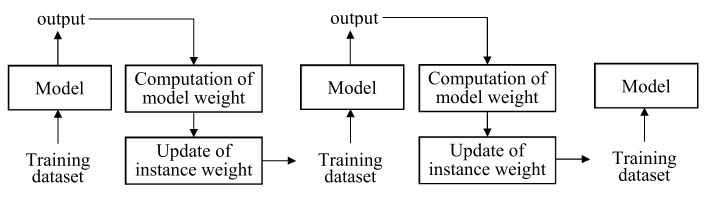
Structure and operation of adaptive boosting.

Boosting is a method of ensemble learning that sequentially learns multiple models such that the model performance gradually increases by correcting the previous model [121,122,123]. The most popular one is adaptive boosting (in short, AdaBoost), as shown in Figure 10 [123]. For classification, we train the first model and identify the misclassified instances in the training dataset. We compute the model weight based on the weights of the misclassified instances and update the weights of the misclassified instances. We then train the second model using the updated instance weights. As such, the second model is better than the first model. In the testing stage, the input is applied to all models simultaneously and their outputs are merged according to the model weights.

## 3. Methods for Performance Evaluation

### 3.1. k-Fold Cross-Validation and Leave-One-Out Cross-Validation

When only a small number of instances are available for a given task, we cannot evaluate the ML performance in a reliable way because of insufficient training and testing data after data partition. To solve this problem, we use k-fold cross-validation, which facilitates multiple trials of training and testing using different datasets [124,125]. We randomly divide the entire dataset into k subsets of equal size and assign one subset to the testing dataset and the remaining k−1 subsets to the training dataset. Then, as usual, we train the model using the training dataset and measure the performance using the testing dataset. We repeat this process k times using each of k testing datasets and obtain k different models and their performance. In this way, we test each instance in the dataset exactly once. Finally, we average the performance of k models and obtain the final performance. For data partitioning in classification, it is desirable to maintain the relative proportions of classes in the training and testing datasets for more generalized modeling and evaluation. This type of partitioning is called a Latin-partition [126].

When the size of the dataset is too small, normal k-fold cross-validation is not feasible because of the small size of the training dataset. We can then use leave-one-out cross-validation (LOOCV) for more reliable modeling [125]. If the number of instances in the entire dataset is D, we use one instance for testing and the remaining D−1 instances for training. By repeating this with different instances for testing, we obtain D different models and compute their average performance for the final performance. LOOCV corresponds to a special case of k-fold cross-validation with k=D, and is an exhaustive approach that divides the original dataset into two datasets with one and D−1 instances in all possible ways. It can be extended to more general leave-p-out cross-validation.

### 3.2. Evaluation Metrics

#### 3.2.1. Confusion Matrix

**Figure 11:**
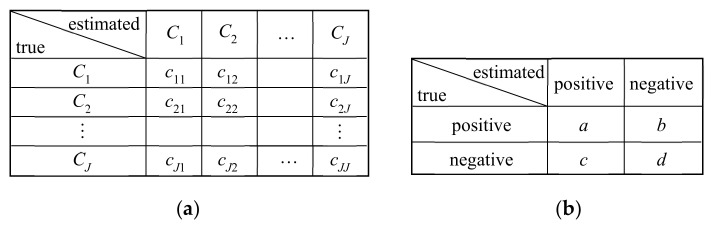
Examples of confusion matrix. (**a**) Multi-class classification; (**b**) Binary classification.

A confusion matrix is a method to express the overall performance of classification as shown in Figure 11, wherein the row represents the ground-truth class and the column represents the estimated class. For example, the confusion matrix in Figure 11a for J-class classification implies that, out of $\sum_{j=1}^{J}c_{ij}$ trials of class $C_{i}$ inputs in the testing dataset, $c_{ij}$ trials are classified as class $C_{j}$. For each class $C_{i}$, $c_{ii}/\sum_{j=1}^{J}c_{ij}$ is the rate of correct classification and called a recall. The average recall across all classes, also called the mean accuracy, is usually used as the overall performance in multi-class classification.

When binary classification into positive and negative for specific diagnosis has the confusion matrix in Figure 11b, the following metrics are used for performance evaluation.

- true positive rate (TPR) = sensitivity = $a/\left(a+b\right)$
- true negative rate (TNR) = specificity = $d/\left(c+d\right)$
- false positive rate (FPR) = 1 − specificity = $c/\left(c+d\right)$
- false negative rate (FNR) = 1 − sensitivity = $b/\left(a+b\right)$

#### 3.2.2. ROC and AUC

In binary classification into positive and negative, the final decision is made by checking if the classifier output y is larger than the threshold λ. Therefore, the choice of λ directly affects the performance, but the confusion matrix does not represent this issue because it only shows the results for a fixed λ. We explain two methods to examine the operation of the classifier as a function of λ.

**Figure 12:**
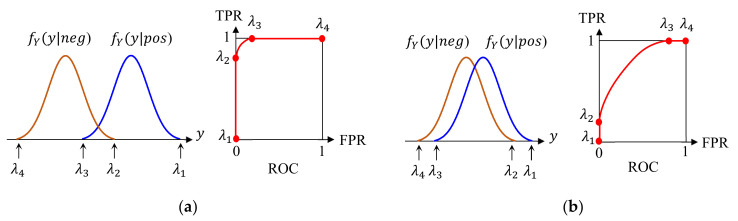
Examples of receiver operating characteristic (ROC) from probability density functions (PDFs) of different overlap. (**a**) Small overlap between two PDFs; (**b**) Much overlap between two PDFs.

Suppose that Y is a random variable for y. We then define two conditional probability density functions (PDF), $f_{Y}(y|pos)$ and $f_{Y}(y|neg)$, wherein each represents the distribution of y when the true class of input is positive and negative, respectively. Then, TPR is the area of $f_{Y}(y|pos$) for y≥λ and FPR is the area of $f_{Y}(y|neg)$ for y≥λ. We plot a curve of “TPR vs. FPR” for different values of λ, and the result is called a receiver operating characteristic (ROC). Different classifiers yield different PDFs, resulting in different ROCs. Figure 12 shows examples of PDFs and ROCs; λ in the PDF and the corresponding point in ROC are shown for better understanding by the readers. We can observe a distinct relationship between the PDF overlap and the shape of the ROC. Hence, without knowing the actual PDF, we can roughly predict how far two classes are separated with respect to y from the shape of the ROC. Finally, the shape of the ROC is quantified by the area under the curve (AUC) in the ROC. AUC ranges from 0.5 to 1.0, and a higher AUC indicates a better classifier.

#### 3.2.3. Coefficient of Determination

The coefficient of determination, which is usually denoted as $R^{2}$, is an evaluation metric for regression that measures the average deviation between the target values and the estimated values. For the n-th instance in this dataset, let the target value be $y_{n}$ and the estimated value be $y_{n}^{\prime }$. Then, the estimation error is $e_{n}=y_{n}-y_{n}^{\prime }$ and $R^{2}$ is defined by Equation (6) for all instances in the testing dataset. $R^{2}$ measures the effectiveness of the estimator in comparison to a baseline estimator that always predicts $y_{n}$ as $\bar{y}$ for all n. A perfect estimator yields $R^{2}=1.0$ as an upper limit, whereas a baseline estimator yields $R^{2}=0.0$. The worse estimator may yield negative $R^{2}$ value.

(6) $$R^{2}=1-\frac{\sum_{n}e_{n}^{2}}{\sum_{n}{\left(y_{n}-\bar{y}\right)}^{2}},\bar{y}=average\left(y_{n}\right)$$

## 4. Examples of THz Imaging and THz-TDS Based on Machine Learning

From an ML perspective, THz applications are characterized by using regulated signals with the same properties within a given task. This nature is not applicable to real-life applications such as speech recognition, wherein speech signals are produced by various people speaking in different ways. Therefore, we can learn a task-specific model without worrying about its robustness to varying signal properties, which suggests the potential of high ML performance. However, this nature also adversely affects the ML performance, because a huge number of training data are not provided. As a result, the model complexity is limited by the size of the available training dataset. Another problem with THz applications is that THz signals often have low spectral and spatial resolution, compared with those captured by commercial microphones and cameras. In addition, they tend to be noisy due to measurement noise and system instability. Therefore, THz applications demand special strategies to deal with low-resolution noisy signals.

Considering these characteristics of THz applications, we explain specific ML processes in representative examples of THz imaging and THz-TDS. The examples were selected to cover various tasks of classification and regression that use different ML techniques for both 1-D signals and 2-D images. As such, the examples show a wide scope of applying ML to different tasks and provide a guideline for designing ML solutions for new tasks. The performance of each example is also summarized. For each example, however, no performance comparison with other methods is provided, because each example considers a specific task with unique system set-up, test samples, and experimental conditions and its baseline performance cannot be defined.

### 4.1. Diagnosis of Cervical Carcinoma

Qi et al. developed a method for the diagnosis of cervical carcinoma using THz-TDS [12]. Cervical tissues consisting of 32 normal and 20 cancerous tissues were obtained and cut into slices of 8 μm thickness. After appropriate treatment of the slices, transmitted THz spectra of the samples were acquired using a THz-TDS system, and the refractive index and the absorption coefficient of each sample were calculated. They were then transformed into other data using various transform methods [12]. Each set of the transformed data was used as input features for classification, and feature reduction was not performed. Two classifiers were built using SVM and partial least squares-discrimination analysis [127].

The classification performance was evaluated based on bootstrap sampling, which is a method of random data sampling from a given dataset with replacement [126]. Each trial of bootstrap sampling resulted in a unique composition of data, and the sampled data were Latin-partitioned into five subsets. One subset was then used for testing and the other four subsets were used for training. The classification results for five different assignments of training and testing datasets were obtained and pooled. Finally, 50 bootstrap samples were generated and the average performance across all bootstrap samples was computed. The SVM classifier using features obtained using both the Savitzky–Golay first derivative [128] and principal component orthogonal signal correction (PC-OSC) [129] had a sensitivity of 88.6% and a specificity of 96.7% [12].

### 4.2. Identification of Materials

Li et al. investigated a classification method to identify unknown material among 14 materials, including six metal-containing materials and eight non-metal materials [43]. The former included zinc, iron, cobalt, cadmium, mercury, and copper, and the latter included tetracene, polyfilm, polyethylene, terephthalic acid, asphalt, trehalose, milk power, and coffee. THz spectra of these materials were obtained from a THz spectroscopic database established by NICT and RIKEN [42]. A total of 107 spectra were examined in [43].

**Figure 13:**
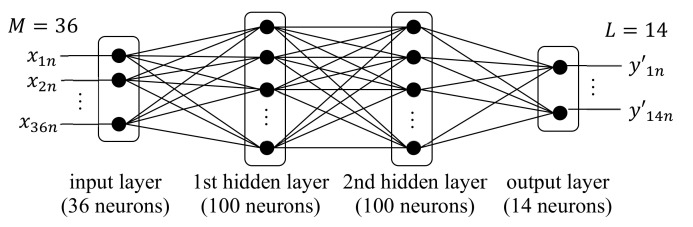
Deep neural network (DNN) structure for classifying 14 materials in [43].

For feature extraction, the approximate entropy (ApEn) of the THz spectrum was computed, which measures the complexity and regularity of a signal using two parameters: the window length W and the filtering level r [130]. W was set to 1, 2, 4, and 8, and for each W, the ApEn values were computed using 36 different values of r defined as $r=k10^{i-4}$, 1≤k≤9, 0≤i≤3 [43]. These 36 ApEns were used as features for each W, and no feature reduction was performed. Four different classifiers were implemented using W=1, 2, 4, and 8, and their performance was compared to empirically determine the optimal W. The classifier was designed using a DNN with two hidden layers, as shown in Figure 13 [43]. Given that the task was 14-class classification (L=14), the output layer had 14 neurons.

The classification performance was evaluated using the LOOCV approach. By comparing the performance for different W, it was determined that the optimal performance was obtained when W=4. In this setting, the overall accuracy for classification into 14 materials was 80.4% [43]. In most cases, metal materials were not misclassified as non-metal materials, and vice versa. When the classification scheme was changed to binary classification into metal and non-metal classes, the accuracy increased to 96.3% [43].

### 4.3. Evaluation of Traumatic Brain Injury

Shi et al. developed a method for automatic evaluation of the degree of traumatic brain injury (TBI) based on THz transmission imaging [15]. Adult male rats with different degrees of TBI were prepared, and the fresh brain tissues were cut into 40 μm thick sections. After appropriate treatment of the tissues, the sectioned biological samples were mounted on a linear motor stage for raster-scan imaging, and the THz transmittance data of the samples were acquired. There were 64 rats with TBI and 16 rats in the sham operation group, and a total of 80 images were acquired with one image for each rat [15]. The degree of TBI was categorized as mild, moderate, and severe TBI, and the number of each category was 18, 27, and 19, respectively. The task was four-class classification, including the sham group.

**Figure 14:**
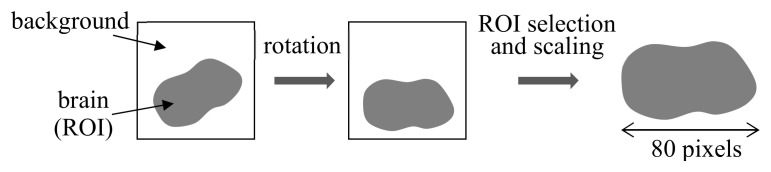
Processing of images prior to feature extraction in [15].

Prior to feature extraction, images were processed to normalize for different sizes and directions. As shown in Figure 14, the region of the brain inside the image, called the region of interest (ROI), was identified by pixel-level thresholding, and rotation rectification was applied to the image to align the image direction [15]. The image was scaled to have the same ROI size of 80 pixels in the horizontal direction. Then, the features of two different aspects were extracted from the ROI in the processed image. First, the minimum, maximum, and mean values of the pixels in each column of the ROI and those in all columns of the ROI were calculated, resulting in 80 × 3 + 3 = 243 features [15]. These features characterize the spatial distribution of pixel values in different classes. Next, the grayscale histogram of pixel values in the ROI was computed with 256 bins, which yielded an additional 256 features [15]. They characterize the statistical distribution of transmittance in the brain region of different classes. In this way, a total of 499 features were extracted from an image.

Feature reduction was performed based on ReliefF, resulting in different numbers of reduced features, from one to 499. Three different classifiers were designed using kNN, SVM, and random forest. Each classifier was independently trained using the LOOCV approach, and the optimal number of reduced features for each classifier that yielded the highest accuracy was empirically determined. Overall, random forest yielded the best performance. Specifically, kNN using all 499 features and k=12 resulted in a mean accuracy of 86.25%, SVM using 141 features yielded a mean accuracy of 83.75%, and random forest with 50 decision trees and 161 features had a mean accuracy of 87.5% [15]. ROC and AUC of one-vs.-other classifications were also computed using the selected optimal number of features for each classifier.

The recognition of mild TBI as a binary classification was also conducted because the accurate identification of mild TBI is the bottleneck in current biological imaging technologies [15]. The performance using kNN, random forest, and SVM is summarized in Table 1 [15].

### 4.4. Quantitative Characterization of Bovine Serum Albumin Thin-Films

Sun et al. studied a regression method to estimate the quantitative characteristics of bovine serum albumin (BSA) thin-films based on THz-TDS [44]. The BSA stock solutions were prepared at 21 different concentrations from 0.5 to 35.0 mg/mL and were pipetted onto each quartz substrate of 0.5 mm thickness to fabricate BSA thin-films of different concentrations [44]. The THz signal of the thin-film was acquired seven times for each sample using THz-TSD system, resulting in 21 × 7 = 147 spectra with 43 frequency bins.

In the pre-processing step, PCA-based denoising was conducted. For each concentration, the scores of seven measurements for the first two principal components were computed, and distinct outliers that were far from the centroid of the seven scores were identified. As a result, 12 outliers were found and the remaining 147 − 12 = 135 spectra were used for machine learning [44]. The raw spectra with 43 frequency bins were used as input features, without additional feature extraction.

A regression model was designed using SVR with a radial basis function kernel. SVR searches for a function from 43 input features to a target concentration value. Once the optimal function in SVR was found, the concentration of the test BSA thin-film was estimated by applying its spectrum to the function. The estimation performance using all 43 frequency bins was evaluated using two schemes. First, LOOCV was used and a coefficient of determination, $R^{2}$, of 0.97272 was obtained [44]. It was also confirmed that there was a strong correlation between the true and estimated values for all concentrations [44]. One of the challenges associated with LOOCV is that instances of the same concentration were included in both the training and testing datasets, which can ease the estimation task. To eliminate this shortcut, the performance based on a hold-out validation scheme was also evaluated. Specifically, all instances from one concentration was held out for testing dataset and the remaining instances from other concentrations were used as the training dataset. This was repeated until each concentration was used once for the testing dataset, and the average performance was computed as the final performance. Then, $R^{2}$ decreased to 0.91651, due to the limited information in the training stage [44].

In addition to normal estimation using all the frequencies, the importance of each frequency in estimating the concentration was investigated using MIC, which measures the relationship between each frequency and the target concentration. It was determined that the most important frequencies were 1.2 THz, 1.1 THz, and 0.5 THz in descending order [44]. The performance was then evaluated based on the given number of the most important frequencies identified using MIC. The three most important frequencies yielded a performance close to that using all 43 frequencies [44]. When more than the three most important frequencies were considered, the performance increment was not significant [44]. This result indicated that the BSA concentration is highly correlated with a few important frequencies in the THz spectra.

### 4.5. Identification of Components in a Mixture

Peng et al. developed a regression method for estimating the concentration of noradrenaline (NE) and N-acetylaspartate (NAA), which are two key diagnostic substances in human brain cells, in a mixture of seven substances [47]. Ten mixture samples of seven substances were prepared and pressed as 13 mm tablets with a thickness of 2 mm. The masses of all the tablets were set to 130 ± 5 mg and the concentrations of NE and NAA were randomly set to the 0–12% range, according to their concentrations in the human brain [47]. The absorption spectra of the samples were then acquired using a THz-TDS system, resulting in 10 spectra with different mixture concentrations of NE and NAA. In this study, the spectrum of an individual substance was not acquired [47].

In the pre-processing stage, the noise and slanted baseline in the acquired spectra were eliminated using a wavelet transform. Using the Daubechies 9 wavelet, the spectrum was decomposed up to six levels [131,132]. Then, the frequency components that represent noise were eliminated, and only useful frequency components were retained at each level. Subsequently, the slanted baseline was estimated using polynomial fitting, resulting in an enhanced spectrum with the baseline removed [47]. This spectrum was used as an input feature for machine learning.

A regression model based on SVR was designed to estimate the concentrations of the target components, NE and NAA, from the input spectrum. In SVR, a Gaussian kernel function was applied to the input to achieve high-dimensional space with improved identification properties, after the empirical selection of the best kernel variables based on performance evaluation. Since there were only 10 available instances in the dataset for the task, model learning and performance evaluation were conducted using repeated trials with different data partitions. Specifically, 10 spectra were randomly divided into five partitions, each with two spectra, and four were used for training and one was used for testing [47]. This process was repeated five times for new random partitions, and the results were pooled for the final performance.

The coefficient of determination, $R^{2}$, and the root-mean-square-error (RMSE) were used to measure the estimation performance. The higher $R^{2}$ and lower RMSE are indicative of a better estimation. For performance comparison, the performance of the neural network (NN) was also measured. Table 2 summarizes the estimation performance of the SVR and NN models [47]. The poor performance in NN was due to the small training dataset, which caused poor learning outcomes of the NN, converging to a local minimum point that was much higher than the optimal point.

### 4.6. Analysis and Inspection of Mouse Liver Injury

Huang et al. investigated a classification method to inspect the degree of mouse liver injury using THz-TDS [16]. Twenty-three female mice were used, and different degrees of liver injury were induced by intraperitoneal injection of 5-FU at 25, 50, 75, and 100 mg/kg per body weight under experimental conditions [16]. Five degrees of liver injury including a placebo group were defined. The liver tissues were cut into slices with a thickness of approximately 350 μm, and THz spectrum was measured at 1–3 points on each slice. In this way, 378 spectra were obtained in total, and each class of degree had 64, 62, 114, 88, and 50 spectra, respectively [16].

In the pre-processing stage, distinct outliers in the spectra due to measurement instability were removed using statistical boxplots, and the spectrum was converted to the absorption coefficient and refractive index, which correspond to the original input features. There were 50 absorption coefficients and 50 refractive indices in the 0.2–0.8 THz range [16]. As the first step of feature reduction, MIC was applied to the original features, and the relationship between each feature and the target liver injury level was analyzed. Among the 100 original features, MIC selected the 40 most important features; the selected absorption coefficients and refractive indices were mainly in the range of 0.2–0.6 THz and 0.2–0.3 THz, respectively [16]. In the second step of feature reduction, PCA was applied to both the 100 original features and the 40 selected features, and the first five principal components were computed for both cases [16]. Then, three different feature sets, namely original, PCA, and (MIC + PCA), were independently used as the final input features.

Two classifiers were designed using random forest and AdaBoost, and two types of classification were considered for each classifier. The first was three-class classification, wherein each class corresponds to injection concentrations of 0, 50, and (75 and 100) mg/kg. Its performance in terms of recall is summarized in Table 3 [16]. In both classifiers using random forest and AdaBoost, the performance was improved in the order of the original, PCA, and (MIC + PCA) features. AdaBoost outperformed random forest when the reduced features were used; the performance relationship was reversed when the original features were used. The second type of classification was five-class classification with the original five classes of liver injury level. As shown in Table 3, the accuracy is lower compared to three-class classification because of the inherent difficulty in discriminating between additional classes. Similar to three-class classification, AdaBoost outperformed random forest in recall when the reduced features were used.

### 4.7. Qualitative and Quantitative Detection of Liver Injury

As a continuation of [16], Cao et al. investigated a regression model to estimate the degree of liver injury in mice [18]. Similar to the process of sample preparation in [16], six groups with different liver injury levels were prepared by injecting 5-FU into mice at concentrations of 0, 10, 20, 30, 40, and 50 mg/kg [18]. There were 26 mouse liver tissues, and the number of liver tissues per group was four or five. The liver tissues were cut into slices of 350 μm thickness, and THz spectrum was acquired for each slice using THz-TSD system. In this way, each group had 85, 77, 76, 79, 78, and 69 time-domain spectra, respectively, and there were 464 spectra in total [18].

As a pre-processing step, the distinct outliers were eliminated using statistical boxplots. Then, 50 refractive indices and 50 absorption coefficients were extracted from each THz spectrum in the 0.2–0.8 THz range [18]. Each set of the 50 values was used as the original input feature. Feature reduction was then conducted using MIC, and the 20 most important features were selected [18]. In addition, PCA was applied to both the original features and the selected features, and the first five principal components were used as the final features [18]. The degree of liver injury was quantified by estimating the concentration of the input sample in the regression model, whereas the true concentration had discrete values of 0, 10, 20, 30, 40, and 50. The estimator was built using random forest.

The estimation performance was measured using both the coefficient of determination, $R^{2}$, and the root-mean-square-error (RMSE). Table 4 shows the results for different feature sets and different feature reduction methods [18]. Feature reduction with both MIC and PCA yielded better performance compared to PCA only, and the highest $R^{2}$ was achieved when using the refractive index after feature reduction with both MIC and PCA.

### 4.8. Recognition of Breast Invasive Ductal Carcinoma

Liu et al. investigated a classification method for the identification of breast invasive ductal carcinoma (IDC) using a transmission THz system [19]. The samples were obtained from 63 patients who were diagnosed with breast IDC. Normal and cancerous breast tissues were retrieved from the samples. The tissue samples were categorized as IDC, normal fibrous, and normal adipose samples. They were cut into slices with an approximate area of 10 mm × 10 mm and a thickness of 1.996 ± 0.496 mm. As a result, 97 IDC samples, 100 normal fibrous samples, and 99 normal adipose samples were obtained [19]. Among them, 68 IDC samples, 70 fibrous samples, and 69 adipose samples were included in the training dataset, and the rest were included in the testing dataset [19]. THz time-domain signals were acquired using a transmission THz-TDS system for all the samples.

The features were extracted from the THz time-domain signal based on the wavelet packet transform (WPT) [130] and energy-to-Shannon entropy ratio (ESER) [133]. WPT was conducted by applying recursive low-pass and high-pass filtering to the original THz signal. For each sub-signal obtained from filtering, the Shannon entropy of the signal samples was computed, which represents the complexity of the signal in a compact form. In addition, the normalized energy of each sub-signal was computed with respect to the original THz signal. Then, ESER was computed based on the ratio of Shannon energy to normalized energy. The Daubechies 1 wavelet and the decomposition level of 10 were chosen after the performance analysis, resulting in 1024 ESER features [19].

The number of features were reduced by applying PCA to the original 1024 features, and many candidates of the number of selected principal components were examined and their performances were compared. Numerous classifiers were considered in this study: (i) SVM with quadratic, cubic, and Gaussian kernels; (ii) kNN with cosine, cubic, and Euclidean distances; and (iii) ensemble methods of bagged trees, subspace discriminant, and subspace kNN. For three-class classification into IDC, fibrous, and adipose groups, the highest classification accuracy of 88.9% was obtained using the ensemble method with subspace kNN (k=10) and 30 principal components [19].

The identification of breast IDC in comparison to normal samples as a binary classification was also investigated. The optimal operation for each model with the highest accuracy was selected, and the performance is summarized in Table 5 [19]. The ensemble model provides the highest performance in terms of both sensitivity and specificity.

### 4.9. Tag Identification

Mitsuhashi et al. investigated radio-frequency identification (RFID) in the THz frequency range and a method was developed to identify tags using the THz transmittance spectrum [45]. Two types of tags were examined: one used a polyethylene (PE) sheet with varying thicknesses and the other used different reagents. For PE tags, rust-proofing zinc spray was coated on both sides of the tag, and tags with thicknesses of 0.5, 1.0, 1.5, and 2.0 mm were prepared. To consider the case of hidden tags, they were inserted into a mail envelope, which caused an attenuation of −5 dB in the THz signal at 1.2 THz [45]. The transmittance THz spectrum of the tags over the frequency range of 1.4–1.5 THz was measured at the center of each tag. In each measurement trial, one spectrum was acquired at one measurement point per tag. For each thickness, the signal was acquired nine times, resulting in 36 spectra in the dataset. Among them, 16 spectra were used for training and 20 spectra were used for testing; the testing dataset contained five instances per thickness [45].

For the reagent tags, three chemicals such as aluminum hydroxide, lactose, and maltose, and mixtures of two chemicals were prepared, resulting in six different kinds of reagents [45]. Considering the application of authenticity tags in leather products, synthetic leather and natural leather with a total attenuation of −50dB at 1.2 THz were used as the shielding materials for the THz signal. The transmittance THz spectrum was measured 5–6 times for each of six different reagents over the frequency range of 0.8–1.6 THz, resulting in 32 spectra in total. Among them, 14 spectra were used for training and 18 spectra were used for testing; the testing dataset contained three instances per reagent [45].

For tag identification, using the acquired spectra as input features, various models such as SVM with Gaussian kernel, kNN with k= 1, 2, 3, and DNN were used. The DNN had three hidden layers with 60, 30, and 14 neurons, respectively, from the input to the output direction [45]. Another SVM, called PCA-SVM, was also designed that used the first two principal components for input features, after applying PCA to the spectra. For both PE tags and reagent tags, all tags were correctly identified, except for the case of PCA-SVM for a 1.0 mm-thick PE tag; misidentification was mainly caused by severe feature reduction, resulting in loss of valuable information [45].

**Figure 15:**
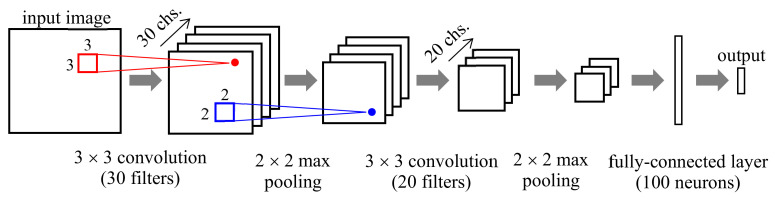
Structure of CNN for real-time tag identification in [45].

In another experiment, real-time tag identification using a combination of multi-wavelength THz waves was investigated at the three frequencies of 1.13 THz, 1.23 THz, and 1.39 THz, corresponding to the peak absorption frequency of each reagent tag under investigation [45]. Images of the detection beam were obtained using a beam profiler. This system facilitates one-shot real-time measurement of the tag. The image was modeled by a CNN shown in Figure 15, and it can identify reagent tags with three single chemicals with 100% accuracy [45].

## 5. Discussion

We examined some results of the recently developed ML-based THz applications. In diagnosing a disease, such as cervical carcinoma, breast IDC, and TBI, the current ML techniques provide a sensitivity of about 90% and a specificity of larger than 95%. In regression tasks for typical biomedical applications, the coefficient of determination larger than 0.9 can be achieved. The performance of multi-class classification depends heavily on the number of classes and the degree of discrimination among classes.

Future research on ML-based THz applications will be focused on designing a specialized ML model that is best suited for a given task. For example, if target information is obscured by attenuation, measurement noise and/or other interfering components, which is often occurred in THz signals acquired from liquids and biomolecules, applying ML in a straightforward way using off-the-shelf models might not guarantee acceptable performance. To design a specialized model for the task, we should first analyze the unique properties and constrains of the signals, which are determined by the THz system in use and the characteristics of test samples, and then investigate how they affect learning behavior in various ML models. Once the final model is designed, we optimize its operation by choosing the optimal hyper-parameters for the task. Because it is not affordable to obtain a large training dataset independently for each task, research on data augmentation suitable for THz signals is also required. In parallel with advances in ML techniques, THz technologies for fast acquisition of high-quality signals will be necessary to improve the performance of task to the level for clinical and commercial applications.

## Figures and Tables

**Table 1:** Recognition performance of mild traumatic brain injury (TBI) in [15].

	kNN	Random Forest	SVM
Sensitivity (%)	77.8	88.9	83.3
Specificity (%)	96.8	95.2	91.9

**Table 2:** Performance of estimating the concentrations of N-acetylaspartate (NAA) and noradrenaline (NE) in a mixture in [47].

	Sample		RMSE			$R^{2}$	
Model		NAA	NE	Average	NAA	NE	Average
SVR		0.40	0.40	0.40	0.98268	0.98287	0.98277
NN		-	-	1.75	-	-	0.69772

**Table 3:** Classification performance of liver injury level in terms of recall (%) in [16].

	Model and Features	Random Forest			AdaBoost		
Task		Original	PCA	MIC + PCA	Original	PCA	MIC + PCA
3-class		70.0	82.5	90.0	67.5	85.0	92.5
5-class		64.3	75.7	82.9	62.3	82.9	88.3

**Table 4:** Estimation performance of liver injury level in [18].

	Features	Absorption Coefficient		Refractive Index	
Measure		PCA	MIC + PCA	PCA	MIC + PCA
$R^{2}$		0.830	0.914	0.824	0.930
RMSE		5.556	4.740	5.629	4.879

**Table 5:** Recognition performance of breast invasive ductal carcinoma (IDC) in [19].

	Ensemble	kNN	SVM
Sensitivity (%)	89.66	89.47	89.65
Specificity (%)	96.67	93.33	94.80

## Data Availability

Data sharing not applicable.
